# On Various Forms of Dyspepsia

**Published:** 1848-05

**Authors:** Abraham Livezey

**Affiliations:** Lumberville, Pennsylvania


*On various forms of Dyspepsia.* By Abraham Livezey, A. M., M. D., Lumberville, Pennsylvania.

The physician, practising in the country, is probably harassed by no class of people so much as by a large family of *foot patients*, who are constantly upon his heels—putting up petitions of a thousand ailments for him to prescribe for,—and for all of which he is expected to give speedy relief, and that too without restricting them as regards their *meat and drink.*

The class to which I allude, embraces a multitude, denominated as *miserable dyspeptics !*

The ordinary symptoms of flatulency, acidity and pyrosis, a constant fulness in the epigastrium,—every particle of food producing a sensation of fulness and heaviness, or a severe pain—gastrodynia—a constant and distressing vomiting of every meal, are cognizant to the merest tyro in medicine.

But there are other marks of indigestion that may escape the young practitioner, and for the alleviation of which, he may exhaust the domain of the materia medica, and even then have the mortification of learning that his patient is having recourse to quack medicines, or else seeking counsel of some senior in the profession.

Some of these are, a troublesome tension of the brain, or of the skin across the forehead, headache, which of all other aches is complained of most frequently, for which the inexperienced physician will, too often, under the hypothetical assumption of hyperæmia in the brain, vainly essay the lancet and the cupping instrument ! And to what end, forsooth ? Plainly to add to that instability of brain, already in a weakened nervous system from irradiated irritations ; from being overtaxed in study or business, from fear of pecuniary losses, or perhaps in merely brooding over their true or imaginary ailments ! The stomach and the bowels are the constant solicitude of the miserly banker who feasts upon a golden table ; of the bustling merchant engrossed in monied transactions ; the deep lawyer ; the hard student, and the ripe scholar. So also with aged persons,—persons whose brains become weaker and weaker by the slow and certain operation of time. These suffer, too, from palpitations of the heart, from giddiness and sensations like fainting with a fear of *falling*, which disposition is most commonly felt upon suddenly raising the head from a procumbent or stooping position. What greater evidence do we want of pure cerebral weakness ? And yet too often are leeches employed to the temples, cups to the nape of the neck, and even the lancet—"that minute instrument of mighty mischief"—is in requisition! Strengthen that debilitated nervous condition, by iron, quinine, nitrate of silver, or other *constitutional* tonic medicines, and energy will be imparted to the corporeal force, and health will be established in the whole "inner man."

Ringing in the ears, sleepless nights, or unpleasant and terrifying dreams, are common complaints among some dyspeptics; others are troubled with a tightness of the throat or chest, and females with a spasmodic action of the œsophagus, with a "stoppage of the wind," which gives them a feeling "as if they had a bell there." In fine, the disease is protean in its character and in its symptoms.

The ordinary prescriptions of bicarb. sod. vel. potass. et zingib., together with the vegetable bitters, cordials, and laxatives, constitute merely a *placebo* mode of treatment, seldom doing more than giving temporary relief; for in all such we maintain that there is a *totality* of derangement, that every part of the constitution of a dyspeptic patient is more or less disordered: in fine, that it is a *constitutional*, not a *local* disease; and hence, in all chronic cases, constitutional remedies are required to overcome the pathological condition. Give then the ferri. præp., quin. sulph., or argent. nitras., each of which acts as a tonic, and apparently sometimes as a sedative, to the nervous system; and thereby improving the tonicity of the organs of digestion.

For the violent spasms, cramps, and pain in the cardiac or epigastriac region, two or three drops of hydrocyanic acid, followed by a bowl of *hot* tea, will give immediate relief, and a continuance of the acid for ten days (i.—ii. gtt. ter. die.) will generally free the patient for months.

A most interesting case came under my care last fall, which had been prescribed for by a medical brother for six months. A delicate female, æt. 17, weight less than fifty, who had been living on one moiety of a hard "water cracker," twice or thrice a day, with a mere sup of cold water for six months, and even this morsel occasioned severe pain, distress, and distension,—complained much of tightness of the throat and upper part of her chest, acid eructations, furred tongue, complete loss of appetite, disordered bowels, œdema of face, feet and stomach, or rather of the inferior portion of the entire chest, and a debility that only enabled her to *totter* from the bedside to her chair. Now, for a long period she had been treated by alkalies and carminatives, various alteratives, vegetable bitters and wine bitters, elixir vitriol, &c., to no effect whatever. Then followed a course of quack medicines, such as Jayne's Alterative, Vermifuge, Balsam, and Sanative pills, &c., all of which left her in the condition above described.

My first prescription was as follows:

℞. Rhei. palmat. pulv. ʒi.
Menth. ppt. fol. "
Potass. Bicarb. "
Sacchar. alb. ℥i.
Aquæ. bullien. ℥iv.

Make an infusion, when cool strain, and add a tablespoonful of best French brandy. S. A dessert spoonful four times daily.

In addition to this she took, fasting, pil. argent. nitrat. gr. $\frac{1}{4}$. In less than a week she could eat two or three crackers, and drink a cup of coffee at a meal, without any inconvenience. To invigorate her system and the condition of her lungs, through a portion of which the respiratory murmur was scarcely audible, and absolute dulness existed under the clavicles, the following was ordered:

℞. Wood Naphtha Rect. ℥i. s. gtt. xx. ter. die.

A spoonful of the former prescription to be taken when any symptoms of acidity manifested themselves. The pills also continued before meals. Naptha at $9\frac{1}{2}$ and $3\frac{1}{2}$ o'clock, and at bed time. At the end of the third week she could walk one hundred yards, and appetite and respiratory murmur much improved.

The œdema now alone remained, which the potas. iod. in solution, v. grs. ter. die. in ten days removed, and the catamenia, after a lapse of six months, was restored in twenty days more by the precipitated carbonate of iron, and the patient was considered perfectly restored in every respect.

Vomiting of meals, gastrodynia, and pain after eating, with eructations, will speedily be remedied by nitrate of silver ($\frac{1}{6}$–$\frac{1}{4}$ gr.)

or hydrocyanic acid. In two cases, where the vomiting returned after the use of the nitrate, the following

℞. Tinct. Cantharid. gtt. x.
Acid. hydrocyan. " i.—ii.

cured them permanently.

In conclusion, if the physician will ring the changes upon some one or other of these remedies, singly or combined, occasionally assisting them by some *symptomatic* medicines, as soda or potassa, for acidity, magnes. usta. et carbo ligni, or rheum, or mush of chopped wheat, to obviate constipation, almost every case will become remediable, without a resort to charlatans and their secret remedies, and then this disease will be stricken from the list of the *opprobia medicorum*, which, as the writer loves his profession, is his earnest desire.

### *Addenda.*

A neighbouring physician succeeded in arresting the vomiting of a case, worse than the one described above, after a lapse of fifteen months, in which the chief symptom was the rejection of *every particle* of nourishment, as well as all drinks,—by small doses of biniod. of mercury. (Five consultations were held over the case.)

A lady, ætat. 86, in my practice, lately fractured both bones of the right leg, which have united perfectly, and she now enjoys the complete use of the same—capable of sustaining her entire weight which I should judge to be 180 pounds at least. She is much drawn by rheumatism, and being unable to recline any length of time in one position, I procured a light portable fracture box, in which the fractured limb was firmly placed, and the whole supported on a double inclined plane, with a loose joint, which enabled her to occupy the sitting position. After a few weeks, the box was removed and we made a strong laced stocking, well splinted with whalebone and laced tight. The fractured bones remained *in situ*, and complete recovery ensued as above stated.

